# Focus on adrenal and related causes of hypertension in childhood and adolescence: Rare or rarely recognized?

**DOI:** 10.20945/2359-3997000000507

**Published:** 2022-08-04

**Authors:** Flávia A. Costa-Barbosa, Rafael B. Giorgi, Claudio E. Kater

**Affiliations:** 1 Universidade Federal de São Paulo Departamento de Medicina da Escola Paulista de Medicina Divisão de Clínica Médica e Divisão de Endocrinologia e Metabolismo São Paulo SP Brasil Divisão de Clínica Médica e Divisão de Endocrinologia e Metabolismo do Departamento de Medicina da Escola Paulista de Medicina da Universidade Federal de São Paulo (EPM-Unifesp), São Paulo, SP, Brasil; 2 Universidade Católica de Sorocaba Ambulatório de Adrenal da Divisão de Endocrinologia da Faculdade de Ciências Médicas e da Saúde da Pontifícia Divisão de Endocrinologia e Metabolismo do Departamento de Medicina da Escola Paulista de Medicina da Universidade Federal de São Paulo (EPM-Unifesp) Sorocaba SP Brasil Divisão de Endocrinologia e Metabolismo do Departamento de Medicina da Escola Paulista de Medicina da Universidade Federal de São Paulo (EPM-Unifesp); Ambulatório de Adrenal da Divisão de Endocrinologia da Faculdade de Ciências Médicas e da Saúde da Pontifícia Universidade Católica de Sorocaba (PUC-Sorocaba), Sorocaba, SP, Brasil; 3 Universidade Federal de São Paulo Departamento de Medicina da Escola Paulista de Medicina Unidade de Adrenal e Hipertensão e Laboratório de Esteroides da Divisão de Endocrinologia e Metabolismo São Paulo SP Brasil Unidade de Adrenal e Hipertensão e Laboratório de Esteroides da Divisão de Endocrinologia e Metabolismo do Departamento de Medicina da Escola Paulista de Medicina da Universidade Federal de São Paulo (EPM-Unifesp), São Paulo, SP, Brasil

**Keywords:** Hypertension, childhood, adrenal

## Abstract

High blood pressure (BP) is not restricted to adults; children and adolescents may also be affected, albeit less frequently. Aside from unfavorable environmental factors, such as obesity and sedentary life leading to early-onset essential hypertension (HT), several secondary causes must be investigated in the occasional hypertensive child/adolescent. Endocrine causes are relevant and multiple, related to the pituitary, thyroid, parathyroid, gonads, insulin, and others, but generally are associated with adrenal disease. This common scenario has several vital components, such as aldosterone, deoxycorticosterone (DOC), cortisol, or catecholamines, but there are also monogenic disorders involving the kidney tubule that cause inappropriate salt retention and HT that simulate adrenal disease. Finally, a blood vessel disease was recently described that may also participate in this vast spectrum of pediatric hypertensive disease. This review will shed some light on the diagnosis and management of conditions, focusing on the most prevalent adrenal (or adrenal-like) disturbances causing HT.

## INTRODUCTION

Hypertension (HT) in infancy is defined as blood pressure (BP) levels above the 95^th^ percentile for age, height, and sex. Although HT is highly prevalent (34%) in adults, children and adolescents are not exceptions to this condition; the prevalence of HT in adolescents and young adults (12-19 years) is approximately 4% in the USA ( 1 ). Both behavioral and environmental factors (obesity, sedentarism) are significant contributors to “primary HT” in infancy and adolescence. According to a recent American Academic of Pediatrics Guideline, workup for secondary causes of HT is not required in children ≥ 6 years who have a family history of HT, are obese, and/or do not have a history or physical examination suggestive of secondary HT and no evidence of end organ damage (moderate recommendation) ( 2 ). Likewise, although some causes of secondary HT are clearly diagnosed by history or clinical findings, others remain obscure ( 3 ).

Adrenocortical hormones, glucocorticoids (GC) and mineralocorticoids (MC), and adrenomedullary catecholamines exert essential effects on the components of BP: intravascular volume, peripheral retention of sodium and fluids, expansion of extracellular volume, hypokalemia, and suppression of plasma renin activity ( 4 ). Activation of this mechanism results from multiple actions along with the renin-angiotensin-aldosterone system (RAAS), mediated by hormone receptors, intracellular factors, enzymatic activity, renal tubule elements, electrolyte transport channels, ATPases, and several others, all encoded by different genes. Pathogenic variants of these genes may result in monogenic causes of HT ( 5 ).

**Figure 1:**
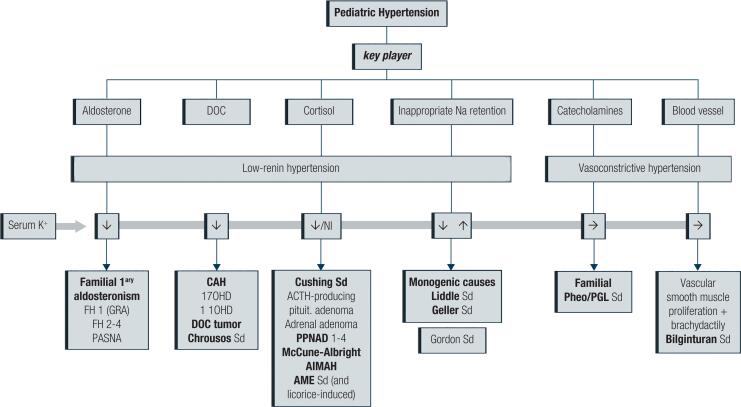
Diagram of causes of pediatric hypertension, classified as low-renin (LRH) and vasoconstrictive hypertension.

Advances in molecular genetics (Next Generation Sequencing era) have allowed the diagnosis of several forms of adrenal-mediated HT. Thus, this review will emphasize monogenic and sporadic adrenal-linked diseases that cause pediatric HT to be didactically discussed. Although several endocrine diseases may be associated with HT in their clinical pictures, such as thyroid and ovarian dysfunctions and prolonged use of certain medications, this review will focus on the most prevalent adrenal (or adrenal-like) disturbances. Accordingly, the subject will be divided by the dominant pathophysiological players: 1) aldosterone; 2) DOC (deoxycorticosterone); 3) cortisol; all three syndromes of excess MC production or activity causing volume expansion and its consequences; 4) catecholamines (familial pheochromocytoma/paraganglioma syndromes); 5) the kidney tubule (gain-of-function mutations of ion transport channels, or kidney tubulopathies simulating adrenal disease); and 6) the blood vessel (syndrome of muscle proliferation of smooth vessels and brachydactyly) (refer to Table 1 and Figure 1 for details).

Serum K^+^: Serum potassium concentration; FH: familial aldosteronism; CAH: congenital adrenal hyperplasia; 17OHD: 17-α-hydroxylase deficiency; 11OHD: 11-β-hydroxylase deficiency; AIMAH: ACTH-independent macronodular adrenal hyperplasia; AME: apparent mineralocorticoid excess; Pheo/PGL: pheochromocytoma/paraganglioma.

**Table 1:** Major hypertensive syndromes in childhood and adolescence, classified according to the key players: aldosterone, deoxycorticosterone (DOC), cortisol, kidney tubule (inappropriate sodium retention), catecholamines, and a blood vessel proliferative disorder. For each condition, the mode of inheritance, clinical features and specific genes affected and pathophysiology are shown, together with presumed levels of serum potassium, renin, aldosterone, DOC, and cortisol

Condition	Mode of inheritance	Genetic mutation	Clinical features	Renin	K_+_	Aldosterone	DOC	Cortisol
**ALDOSTERONE AS KEY PLAYER**								
**PRIMARY ALDOSTERONISM (PA)**								
Familial hyperaldosteronism (FH) type I (glucocorticoid-remediable aldosteronism)	AD	Hybrid *CYP11B1/CYP11B2*	Early on set PA, family history of strokes in young age	↓	↓	↑	→	→
FH type II	AD	*CLCN2*	Early onset HT, BAH	↓	↓	↑	→	→
FH type III	AD	*KCNJ5*	Early onset familial PA	↓	↓	↑	→	→
FH type IV	AD	*CACNA1H*	Early onset familial PA	↓	↓	↑	→	→
Primary aldosteronism with seizures and neurologic abnormalities (PASNA) (type V?)	AD	*CACNA1D*	Early onset familial PA, seizures	↓	↓	↑	→	→
**DEOXYCORTICOSTERONE (DOC) AS KEY PLAYER**								
**CONGENITAL ADRENAL HYPERPLASIA**								
Deficiency of 11-β-hydroxylase	AR	*CYP11B1*	46XX DSD, precocious pubarche boys, HT	↓	↓	↓	↑	↓
Deficiency of 17-α-hydroxylase	AR	*CYP17A1*	46XY DSD, sexual infantilism in glirs, HT	↓	↓	↓	↑	↓
Chrousos syndrome (generalized glucocorticoid resistance)	AD	*NR3C1*	HT, hyperandrogenism, pseudoprecocious puberty, hypoglcemia	↓	→ ↓	→ ↓	↑	
DOC-producing tumor			No cases in pediatric population	↓	↓	↓	↑	→
**CORTISOL AS KEY PLAYER**								
**CUSHING SYNDROME**								
Cortisol-producing adrenal tumor				↓	→ ↓	→ ↓	→	↑
Adrenocortical carcinoma	AD	*TP53*		↓	→ ↓	→ ↓	→	↑
AIMAH	Smu	*GNAS1*	Cushing	↓	→ ↓	→ ↓	→	↑
	AD, Smu	*ARMC5*		↓	→ ↓	→ ↓	→	↑
ACTH-secreting pituitary adenoma	?	*USP8*		↓	→ ↓	→ ↓	↑
Carney complex/syndrome (type I)	AD	*PRKAR1A*		↓	→ ↓	→ ↓	→	↑
PPNAD1	AD	*PRKAR1A*	Skin pigmentation, myxomas, pituitary tumor	↓	→ ↓	→ ↓	→	↑
PPNAD2	AD	*PDE11A(A1-3)*		↓	→ ↓	→ ↓	→	↑
PPNAD3	AD	*PDE8B*		↓	→ ↓	→ ↓	→	↑
PPNAD4	AD	*PRKACA*		↓	→ ↓	→ ↓	→	↑
McCune-Albright syndrome		*GNAS*	Fibrous dysplasia, café-aut lait pigmentation pseudoprecocious puberty					
AME - Apparent Mineralocorticoid Excess Sd.	AR	HSD11B2	Low birth weight, failure to thrive, polyuria, polydipsia, muscle weakness	↓	↓	↓	→	→
**SYNDROMES OF INAPPROPRIATE SALT RETENTION**								
Geller syndrome	AD	*NR3C2*	Early onset HT exacerbated by pregnancy	↓	↓	↓	→	→
Liddle syndrome (type I)	AD	*SCNN1B*		↓	↓	→	→
Liddle syndrome (type II)	AD	*SCNN1G*	Early onset severe HT, metabolic alcalosis	↓	↓	↓	→	→
Liddle syndrome (type III)	AD	*SCNN1A*		↓	↓	↓	→	→
Gordon syndrome	AD			↓		→ ↓	→	→
(pseudohypoaldosteronism type II)	AD	*WNK4*		↓		→ ↓	→	→
	AD	*WNK1*	Short stature, hyperkalemic and hyperchloremic metabolic acidosis	↓		→ ↓	→	→
	AR or AD	*KLHL3*		↓		→ ↓	→	→
	AD	*CUL3*		↓		→ ↓	→	→
**CATECHOLAMINES AS KEY PLAYERS**								
Familial pheochromocytoma	AD	*KIF1B*		→	→	→	→	→
	AD	*SDHB*						
	AD	*TMEM127*						
	AD	*VHL*	HT, palpitations, headache, sweating, abdominal mass, incidental finding, family screening					
	AD	*GDNF*						
	AD	*RET*						
	AD	*SDHD*						
	AD	*MAX*						
**BLOOD VESSELS AS KEY PLAYERS**								
Bilginturan syndrome (hypertension and brachydactily syndrome)	AD	*PDE3A*	Early onset HT, short stature Brachydactyly	→	→	→	→	→

AD: autosomal dominant; AR: autosomal recessive; Smu: somatic mutation; AIMAH: ACTH-independent macronodular adrenal hyperplasia; PPNAD: primary pigmented nodular adrenocortical disease.

## ALDOSTERONE AS A KEY PLAYER ( TABLE 1 ; FIGURE 1 )

### Syndromes of aldosterone excess Primary aldosteronism (PA)

PA is an autonomous secretion of aldosterone, i.e., renin-angiotensin-independent. In adults, PA is the most common cause of secondary HT, leading to cardiovascular damage and high mortality risk. The leading causes are aldosterone-producing adenoma (APA) and bilateral adrenal hyperplasia (BAH), accounting for 90%-95% of cases. The remaining 5%-10% are familial forms with autosomal dominant inheritance, predominantly affecting young people.

Over the last 10-15 years, knowledge of the genetic basis of PA has allowed the identification of Mendelian forms of PA, which are highly prevalent in children and adolescents ( 6 – 8 ).

In brief, the diagnostic management of PA comprises three steps:

- *Screening* : PA is biochemically suspected by an increased ratio of plasma aldosterone concentration (PAC) to plasma renin activity (PRA). A cutoff of 27 for this PAC:PRA ratio (ARR) ( 9 ) (with PAC ≥ 12 ng/dL and suppressed PRA, in ng/mL/h) is highly sensitive (89.8%) and specific (98.2%) ( 10 , 11 )
- *Confirmation* : Lack of response to suppressive maneuvers confirms autonomous aldosterone secretion ( 11 ). Saline infusion, oral sodium load, furosemide, captopril test, or fludrocortisone administration can be used ( 9 – 11 ); the choice depends on service experience rather than test accuracy. Confirmatory tests are usually unnecessary if ARR is > 40 ( 10 ), PRA is suppressed, and hypokalemia is present ( 11 ).
- *Subtype differentiation* : Computerized tomography (CT) with an adrenal protocol initially excluding aldosterone-producing carcinoma. As adrenal incidentaloma (AI) on CT increases with age, favoring false-positive diagnosis of APA or BAH, selective adrenal vein sampling (AVS) is considered the gold standard for differentiating unilateral from bilateral disease. However, in younger patients (<35 years), presenting with a typical unilateral adenoma (>1 cm), hypokalemia, and increased levels of PAC (30 ng/dL), AVS does not need to be performed ( 9 , 11 , 12 ). Additionally, less accurate noninvasive tests (postural stimulation test and aldosterone precursor measurement) can be applied in adults if AVS is unavailable ( 10 , 11 , 13 , 14 ). However, no precise cutoffs of these tests have yet been established in the pediatric population.

#### Familial aldosteronism ( Table 1 )

##### Type I familial hyperaldosteronism (FH1) or glucocorticoid-remediable aldosteronism (GRA)

FH1 results from unequal crossing over between two highly homologous genes (94%), *CYP11B1* and *CYP11B2* . The former encodes 11-b-hydroxylase, which is expressed in zona fasciculata (ZF) and controlled by ACTH, and the latter encodes aldosterone synthase in zona glomerulosa (ZG) under angiotensin and potassium regulation. The mutated chimeric gene comprises the regulatory sequences of *CYP11B1* fused to the coding region of *CYP11B2* , leading to abnormal expression of aldosterone synthase in the ZF, which is dependent on ACTH ( 15 ).

FH1 usually manifests before 20 years of age, and its prevalence is approximately 3% of pediatric HT ( 7 ). Although HT is moderate to severe in most cases, normotensive individuals have been described ( 7 ). Affected patients may have growth and development defects, an increased risk of cerebrovascular disease, and fatal brain hemorrhage before 40 years of age. Patients with FH1 have low renin, increased PAC, hypokalemia (in particular after the use of nonpotassium-sparing diuretics) ( 7 , 16 ), and the presence of hybrid steroids 18-hydroxycortisol (18OHF) and 18-oxocortisol (18-oxoF); imaging studies are compatible with BAH ( 7 ). Molecular identification of the *CYP11B1/CYP11B2* gene by extended polymerase chain reaction (PCR) can confirm the disease. Therapy with long-acting GC may reduce ACTH, but the lowest possible dose that normalizes BP and K should be used. Iatrogenic Cushing's syndrome and impaired linear growth are associated with overtreatment with GC. If BP remains uncontrolled, MC antagonists, spironolactone (SPL), or eplerenone, are necessary. The latter is preferred in children to avoid the common antiandrogenic effects of SPL.

##### Type II familial hyperaldosteronism (FH2)

The recent description of a gain-of-function mutation in the *CLCN2* gene, located on chromosome 3q27.1, has been associated with familial early-onset PA ( 17 ). *CLCN2* mutations lead to efflux of chloride on the ZG cell membrane, favoring continuous aldosterone release. Fernandes-Rosa and cols. also described a de novo mutation in a 9-year-old girl with severe HT, hypokalemia, increased PAC, and low PRA ( 18 ). The phenotypic presentation of FH2 is variable and indistinguishable from sporadic PA, with uni- or bilateral lesions on CT ( 19 ). FH2 has been identified in 10% of young patients with PA ( 17 , 18 ).

##### Type III familial hyperaldosteronism (FH3)

In 2008, Geller and cols. described a family with severe early-onset HT and hypokalemia unresponsive to conventional therapy. They had increased PAC and 18OHF, and 18-oxoF suppressed PRA, which was not controlled by dexamethasone administration. Interestingly, gross macronodular hyperplasia was observed after bilateral adrenalectomy ( 20 ). Next-generation sequencing (NGS) permitted the identification of the first germline mutation in the *KCNJ5* gene, located on chromosome 11p24, which encodes the potassium channel GIRK4 (Kir3.4) ( 6 ). Other studies have shown PA patients with mild HT among FH3 families ( 7 , 21 ). Phenotypic imaging shows predominantly bilateral lesions (macronodular hyperplasia) ( 9 , 11 ). This subtype is rare, with an estimated prevalence of less than 0.5% of PA and 8% of family PA forms ( 7 ).

##### Type IV familial hyperaldosteronism (FH4)

Germline mutations in *CACNA1H* (at chromosome 16p13) ( 22 , 23 ), which encodes the alpha subunit of the voltage-dependent T-type calcium channel Cav3.2 ( 23 ), have been described in children with PA. Although there are no abnormalities in imaging studies, micronodular adrenal hyperplasia has been observed on histology ( 7 , 22 ). Some patients may also manifest the autism spectrum, epileptic disorders, chronic pain, and developmental disorders ( 7 ).

##### Primary Aldosteronism with Seizures and Neurological Abnormalities (PASNA)

De novo mutations in *CACNA1D* , which encodes the alpha-1 subunit of the voltage-dependent Ca2+ L-type Cav1.3 channel, were identified in two children with PA with no adrenal abnormalities ( 24 ). All genetic variations were gain-of-function, facilitating channel opening at low voltages. In these patients, seizures and neurological abnormalities have been described as being associated with PA ( 7 ).

## DEOXYCORTICOSTERONE AS A KEY PLAYER ( TABLE 1 ; FIGURE 1 )

### Syndromes of DOC excess Congenital adrenal hyperplasia (CAH) ( Table 1 ; Figure 2 )

**Figure 2:**
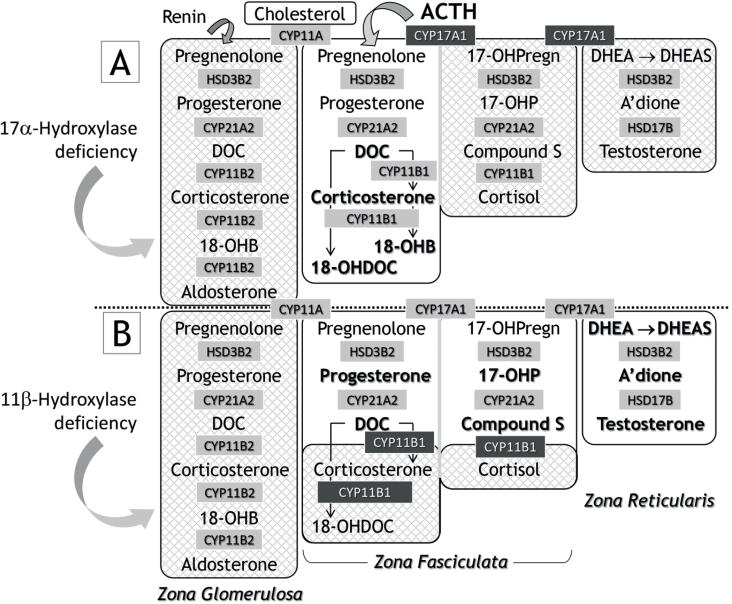
Biosynthesis of adrenocortical steroids in the two hypertensive forms of congenital adrenal hyperplasia: deficiencies of 17-α-hydroxylase (Panel A) and 11-β-hydroxylase (Panel B).

#### 17-α-hydroxylase deficiency (17OHD)

17OHD has an autosomal recessive inheritance mode caused by mutations in *CYP17A1* mapped to chromosome 10q24.3. This gene encodes the expression of 17-hydroxylase, which catalyzes two sequential reactions: 17-hydroxylation of pregnenolone, progesterone, and cleavage of the side chain of the steroid molecule at position 17,20 (lyase activity). The steroid products androstenedione (andro) and dehydroepiandrosterone (DHEA) are immediate precursors of adrenal and gonadal androgens and estrogens. Impaired steroids produce a typical female phenotype (46XY DSD - a disorder of sex development) and absent Müllerian structures. Both XX and XY may present with hypergonadotropic hypogonadism (HH), lack of development of secondary sex characteristics (absence of pubic and axillary hair), primary amenorrhea, eunuchoid habitus, and bone mass impairment ( 25 – 27 ). Moreover, hydroxylation at the carbon 17 position is critical for the formation of cortisol. Thus, the non17-hydroxylated pathway of ZF (17-deoxysteroids) is fully activated by ACTH overproduction, resulting in high concentrations of deoxycorticosterone (DOC), corticosterone (B), 18-OHDOC, and 18-OHB ( 25 , 28 ) ( Figure 2A ). DOC excess is responsible for salt and fluid retention, HT, hypokalemia, and PRA suppression, which restrains the formation of ZG steroids. Interestingly, increased B levels may provide sufficient GC activity to compensate for the chronic state of hypocortisolism ( 25 ).

In Brazil, 17OHD is the second leading cause of congenital adrenal hyperplasia (CAH), accounting for 5%-7% of cases ( 29 ). The presence of founder mutations of Spanish and Portuguese ancestry during colonization, in addition to the wide miscegenation with native indigenous and black Africans during the slavery period, might have contributed to this higher prevalence ( 30 ). Interestingly, Fontenele and cols. described that more than 90% of patients with 17OHD received up to two incorrect diagnoses before the final diagnosis, confirming that 17OHD remains highly underdiagnosed ( 31 ). More than 130 mutations have been detected thus far, but W406R is the most prevalent in Brazil (50%), followed by R362C (approximately 30%) ( 32 – 34 ). HT starts during infancy and is difficult to control, predisposing patients to early renal and cardiovascular outcomes. Increased levels of DOC, B, 18OHB, 18OHDOC, ACTH, LH, FSH, and progesterone but low levels of sex steroids and aldosterone are laboratory hallmarks ( Figure 2A ). Hypokalemia is also common ( 25 , 35 ). The treatment basis of 17OHD is GC supplementation. HT and hypokalemia are readily corrected but, in some cases, may require the addition of SPL or other antihypertensives. Patients of both sexes present female social gender and should receive estrogen therapy from puberty and adult ages. Orchiectomy is also mandatory in 46XY females.

#### 11-β-hydroxylase deficiency (11OHD)

11OHD is generally considered the second most common cause of CAH (5%-8%), except in Brazil and possibly China, where 17OHD is second ( 36 ). Similar to other CAHs, its mode of inheritance is autosomal recessive. In the classic form, defective 11-hydroxylase activity results in a lack of 11-hydroxylation of 11-deoxycortisol (S), resulting in increased S and DOC levels, PRA suppression, and excessive androgen production ( Figure 2B ). Moreover, aldosterone production is reduced in ZG due to PRA suppression resulting from DOC excess; hypokalemia is also present. 11OHD is a 46XX DSD in which girls present variable degrees of genital virilization. In boys, excess androgens lead to penile enlargement, precocious pubarche and puberty, and adrenal rests in the testicles ( 36 ). Mild to moderate HT is present in up to 65% of patients at diagnosis and occurs at birth or soon after ( 37 ). There is no clear correlation between DOC levels and HT or virilization. Because 17-hydroxyprogesterone (17OHP) levels may be moderately increased, several 11OHD patients can be misdiagnosed as 21-hydroxylase Deficiency (21OHD) if S and DOC are not assessed. In this scenario, 21-deoxycortisol (21DF) measurement, which results from 11-beta hydroxylation of 17OHP, is helpful to differentiate both CAH forms ( 38 ). While 21DF levels are increased in 21OHD, their levels are undetectable in 11OHD ( 38 ). Like any form of CAH, treatment involves continuous use of GC to decrease ACTH stimulation of the adrenal cortex, suppressing androgen and DOC excess and their consequences. Over time, HT may become refractory to GC therapy, requiring the introduction of SPL and, occasionally, amiloride and calcium channel blockers ( 36 ).

### Generalized glucocorticoid resistance (Chrousos syndrome) ( Table 1 ; Figure 1 )

Chrousos syndrome is a rare autosomal dominant disease characterized by insensitivity to GC due to mutations in the *NR3C1* gene located on chromosome 5q31 ( 39 ). Thus, increased ACTH levels lead to adrenal hyperplasia and overproduction of adrenocortical steroids (MCs, cortisol, DOC, B, and adrenal androgens). Consequently, the main phenotypic features are HT, hypokalemia, hyperandrogenism, increased cortisol, and ACTH in the absence of Cushing's manifestations ( 39 , 40 ). The treatment goal is to suppress ACTH with small doses of dexamethasone or the use of MC antagonists ( 40 ).

### DOC-producing tumor

Pure DOC-secreting adrenal tumors are rare and have been reported occasionally. No cases have been reported in the pediatric population. However, DOC excess (and mineralocorticoid manifestations) may be part of the steroid admixture produced by an adrenocortical carcinoma.

## CORTISOL AS A KEY PLAYER ( TABLE 1 ; FIGURE 1 )

### Syndromes of cortisol excess Pediatric Cushing syndrome (CS)

The leading cause of pediatric CS is exogenous exposure to synthetic GC. Regarding the endogenous source, hypercortisolism can be divided into ACTH-dependent causes (ACTH-producing pituitary adenoma and ectopic ACTH secretion) and ACTH-independent causes (adrenal adenoma or carcinoma) ( 41 ). Males are predominantly affected during early childhood, whereas girls are affected at later ages ( 41 ). HT results from the interaction of several pathophysiological mechanisms that regulate plasma volume, peripheral vascular resistance, and cardiac output. Regardless of the cause of CS, the ability of 11HSD2 to inactivate F may be compromised, allowing it to access the MC receptor and reproduce aldosterone actions ( 42 ). Thus, regardless of the specific treatment for CS, SPL or eplerenone use may be necessary to minimize the long-term consequences of this disease ( 42 ).

#### Adrenocortical carcinoma (ACT)

ACT is a relatively rare disease in developed countries (e.g., the annual incidence in the United States is approximately 0.3 cases/million), and the incidence in southwest Brazil is approximately 4.2 cases/million/year. It occurs at any age, with a bimodal distribution: a first peak occurring before 5 years of age and a second between the fourth and fifth decades ( 43 ). This unexpectedly high prevalence is mainly due to genetic disorders, such as Li-Fraumeni syndrome (LFs) ( 43 ), which is characterized by germline mutations in *TP* 53, a tumor suppressor located on chromosome 17p13.1. Other phenotypic features of LFs may be sarcomas, osteosarcomas, breast carcinoma, brain tumors, leukemia/lymphoma, adenomas, and adrenocortical carcinomas ( 44 ). Additionally, mutations of the β-Catenin Gene ( *CTNNB1* ), a prevalent cause of ACT in adults and related to poor prognosis ( 44 ), are not commonly detected in childhood ( 45 ). CS and mineralocorticoid HT are the most frequent features. Due to excessive adrenal androgens, early pubarche, virilization (clitoromegaly, penis enlargement, acne, hirsutism, increased muscle mass), irritability, weight gain, altered voice timbre, and short stature may also be evident. Increased levels of DHEA-sulfate (DHEAS) in the presence of the above symptoms are imperative to investigate ACT.

#### ACTH-secreting pituitary adenoma (Cushing's disease)

Cushing's disease (CD) is the most frequent cause of endogenous CS in childhood (after five years of age) and adolescence. The average age at presentation is 14.1 years ( 46 , 47 ). An ACTH-secreting pituitary adenoma may lead to classic hypercortisolism, but hyperandrogenism may also occur by stimulating the adrenal reticular zone. Virilization with pseudo precocious puberty and increased andro, testosterone, and DHEAS are common. As mentioned above, due to the saturation of renal 11HSD2 from excessive cortisol production, HT and hypokalemia are also present. Transsphenoidal surgical excision of the adenoma is the recommended therapy. Cure occurs in approximately 75% of large centers ( 48 ). Other therapeutic options include radiotherapy, steroid inhibitors, and bilateral adrenalectomy in rare cases ( 48 ).

#### Carney syndrome

Carney complex (CNC) is a rare multiple neoplasia syndrome inherited in an autosomal-dominant manner caused by loss-of-function mutations of the PRKAR1A gene located at 17q22-24, which encodes the regulatory subunit type I alpha of protein kinase A (PKA) ( 49 ). CNC is associated with lentigines, primary pigmented nodular adrenocortical disease (PPNAD), and various endocrine and nonendocrine tumors (cardiac and breast myxomas). GH-producing adenomas (which also secrete small amounts of PRL) have been reported with increased frequency ( 50 ). PPNAD is a cause of ACTH-independent CS, causing HT with low renin levels. Despite cases described in children aged 3 years, the peak incidence is between the second and third decades ( 49 ).

#### McCune-Albright syndrome (MAS)

MAS is characterized clinically by the classic triad of polyostotic fibrous dysplasia, cafe-au-lait skin pigmentation, and peripheral precocious puberty. However, it is clinically heterogeneous and can include various other endocrinologic anomalies, such as thyrotoxicosis, acromegaly, and CS ( 51 ). This disease is associated with early embryonic postzygotic somatic activating mutation of the Gs protein's alpha subunit (Gsα protein, encoded by the *GNAS1* gene) ( 52 ). Gsα proteins can also stimulate β2 adrenergic receptors in the cardiovascular system. However, no complications associated with the hyperfunction of β2 adrenergic receptors have been reported in patients with MAS. Mild HT or arrhythmia is associated with hyperthyroidism or hypersecretion of GH in MAS ( 53 ).

### Apparent mineralocorticoid excess syndrome (AMES)

AMES results from the deficiency of 11-β-hydroxysteroid-dehydrogenase type 2 (11HSD2), an enzyme expressed predominantly at nephron distal tubules and collector MC receptor, colon, salivary glands, and placenta. This enzyme converts cortisol (F) into its inactive metabolite, cortisone (E), preventing the activation of the MC receptor. Both PAC and F can activate the MC receptor, but the latter has 1,000-fold higher concentrations than the former steroid ( 54 ). Thus, AMES results in excessive renal exposure to F, producing a state of MC hyperactivity ( 4 ). However, the hypothalamus-pituitary-adrenal axis remains intact, precluding a hypercortisolism phenotype.

AMES is an autosomal recessive disease caused by *HSD11B2* gene mutations (chromosome 16q22), which encode the 11HSD2 enzyme ( 54 ). In the more severe form, type 1 AME (null enzyme activity), patients may be symptomatic during the neonatal period, with low weight, short stature, severe HT, metabolic alkalosis, and muscle weakness. Hypokalemic nephropathy causes nephrocalcinosis, polycystic kidneys, and nephrogenic diabetes insipidus. Mortality is higher than 10% due to cardiovascular diseases in most cases. Type II AMES mutations result in partially decreased 11HSD2 activity. Thus, symptoms start during later adolescence or adulthood in a less severe presentation ( 5 , 55 ). Interestingly, low PAC, DOC in the normal or lowest range, hypokalemia, metabolic alkalosis, and PRA suppression may make the differential diagnosis difficult. However, F and E metabolites are crucial to define AME when performing ratios: tetrahydrocortisol + 5a-tetrahydrocortisol: tetrahydrocortisone (THF + 5aTHF/THE). An increase in 5aTHF/THF and a decrease in THF + 5aTHF/F denotes an A ring reduction impairment. Additionally, free urinary (UF) ratios, such as the UFF:UFE ratio, have good accuracy for AME diagnosis ( 55 ).

Certain conditions may induce AMES, such as excessive use of licorice, grapefruit, and carbenoxolone. These compounds have high amounts of glycyrrhetinic acid, a potent competitive inhibitor of the renal 11HSD2 enzyme ( 54 ). Dexamethasone 1.5 to 2 mg/day suppresses cortisol and normalizes BP and potassium levels in 7-10 days in approximately 60% of cases. SPL, amiloride, and triamterene are complementary options ( 4 , 5 ).

## CATECHOLAMINES (EPINEPHRINE/NOREPINEPHRINE) AS KEY PLAYERS ( TABLE 1 ; FIGURES 1 AND 3 )

### Syndromes of pheochromocytoma/paraganglioma Familial pheochromocytoma/paraganglioma (PPGL) syndromes

**Figure 3:**
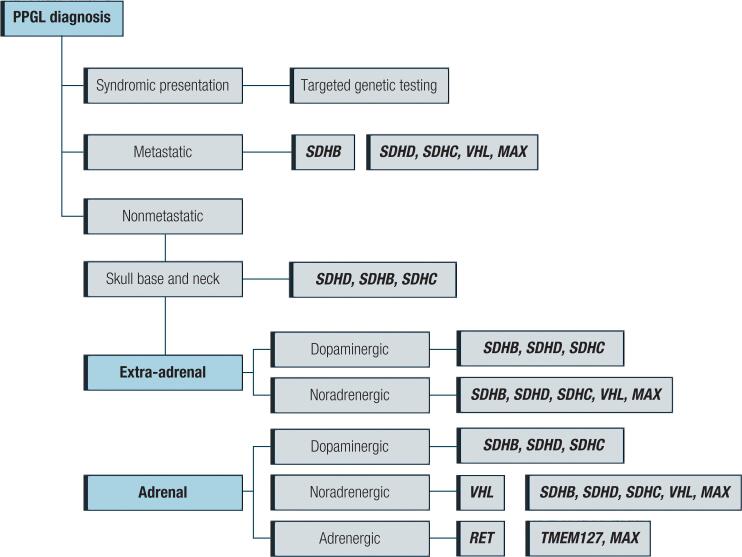
Algorithm for genetic testing in patients with a diagnosis of PPGL (pheochromocytoma/paraganglioma syndrome).

In the pediatric population, PPGL is considered a rare cause of secondary HT (0.5%-2.0%). However, when the diagnosis is delayed, mortality rates are high ( 56 ). Initially, PPGL was considered sporadic in 90% of the cases. However, after NGS studies, up to 35% of PPGLs are due to germline mutations ( 57 ). More than 15 genes have been described thus far, but genetic syndromes commonly associated with PPGL are multiple endocrine neoplasia type 2 (MEN-2), Von Hippel-Lindau disease ( *VHL* ), and neurofibromatosis type 1 (NF1). Then, an extended genetic evaluation is mandatory in the pediatric population ( 57 ) ( Figure 3 ). In a Brazilian cohort, *VHL* was the most prevalent ( 58 ). PPGL can be seen between 1 and 11 years of age, and clinical manifestations are variable. Approximately 60 to 90% of pediatric cases have sustained HT ( 59 ). Orthostatic hypotension, spells and seizures were also observed. Because of the hypercatabolic state, children may experience growth retardation and failure to thrive ( 14 ). Mass spectrometry metanephrine level measurement (plasma or urinary in 24 h) ( 57 ), followed by anatomical imaging tests, CT or magnetic resonance (MRI), and metaiodobenzylguanidine (MIBG) complete the PPGL diagnosis ( 57 ). Surgical removal of the tumor is imperative. However, preoperative management is crucial to prevent intraoperative complications. Alpha-blockers (hypotensive effect, promotion of vasodilation, and volume restoration) must be prescribed at least 2-4 weeks before the procedure ( 56 , 57 ).

## THE KIDNEY TUBULE AS A KEY PLAYER ( TABLE 1 ; FIGURE 4 )

**Figure 4:**
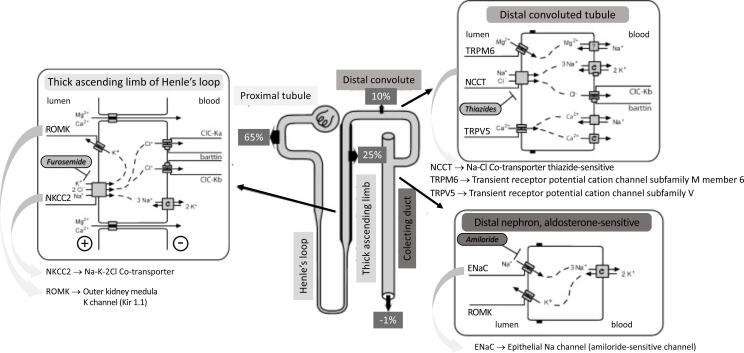
Simplified illustration of the renal tubules, denoting areas (membrane receptors, channels and ions transport) where specific mutations affect sodium transport, promoting inappropriate sodium retention and subsequent hypertension. The percentage of reabsorbed sodium in the different nephron portions is also represented. See Item 5 (The kidney tubule as a key player) for a better comprehension.

### Syndromes of inappropriate salt retention Renal tubulopathies mimicking adrenal disorders

#### Geller syndrome

Geller syndrome is an autosomal dominant disease caused by constitutive activation of the MC receptor due to a functional mutation in the *NR3C2* gene (chromosome 4q31) ( 5 , 60 ). Although there are no natural ligands for the receptor, progesterone and SPL, which are physiological antagonists, start to act as agonists. Thus, HT worsens during pregnancy, a period of physiologically increased progesterone levels. Additionally, E acquires the ability to activate the receptor in this syndrome, leading to severe HT in infancy or adults. PAC and PRA are low or suppressed, but potassium levels remain normal ( 5 ). There is no specific therapy defined for nonpregnant individuals; however, premature delivery is a feasible option in pregnancy owing to high maternal-fetal risk ( 5 ).

#### Liddle syndrome

Liddle syndrome is a rare autosomal dominant disease due to activating mutations in the *SCNN1A* , *SCNN1B* , and *SCNN1G* genes, which code the α, β, and γ subunits, respectively, from the epithelial sodium channel (ENaC), also called the amiloride-sensitive channel. These mutations increase the activity of ENaC and promote sodium retention at distal nephrons and collector tubules, regardless of the presence of aldosterone ( 61 ). Early-onset HT is typical in LS, starting as early as 2 years of age. In the most extensive series of cases, the average age of onset of HT was 15.5 ± 3.3 years ( 62 ). A systematic review revealed that HT is a feature in 92.3% of patients ( 61 ). Liddle described this condition as primary pseudohyperaldosteronism because, although severe HT, hypokalemia, metabolic alkalosis, and suppressed PRA may suggest PA status, PAC is low. Then, LS's suspicion should be on the clinical picture of severe HT since childhood, associated with a remarkable family history. Amiloride or triamterene (ENaC inhibitors) are therapeutic choices. MC receptor antagonists, such as SPL, should not be used ( 62 , 63 ).

#### Gordon syndrome (pseudohypoaldosteronism type II) ( Figure 4 )

Type II pseudohypoaldosteronism (PHA II), also known as Gordon syndrome (GS), is a rare autosomal dominant disease with low renin HT ( 64 ). Five subtypes of PHAII have been described, designated A to E. Type IIA has been associated with chromosome region 1q31-q42 with no gene yet identified, PHAII-B with specific variations in the *WNK4* gene (17q21), and PHAII-C by mutations in the *WNK1* gene (12p12.3.). Finally, germline variations in *KLHL3* (5q31.2) and *CUL3* (2q36) are related to PHAII-D and E, respectively ( 65 and references therein, 66 , 67 ). These genes are involved in a complex multiprotein system that regulates electrolyte transport in the distal nephron. Patients with GS present marked hyperkalemia and a risk of cardiac arrhythmias ( 68 ). Metabolic acidosis (specifically type IV renal tubular acidosis) with preserved renal function and normal or low PAC have also been described ( 64 ).

WNK4 (whose function is reduced by WNK1 and other factors) is a negative regulator of the thiazide-sensitive Na-Cl cotransporter (NCCT) in the distal convoluted tubule (DCT). KLHL3-CUL3 E3 ubiquitin ligase regulates the levels of WNK1 and WNK4. Pathogenic variations of all four genes result in increased NCCT activity in the DCT, leading to the PHAII phenotype. Then, excess sodium and chloride reabsorption is associated with volume expansion, HT, and hyperchloremia. On the other hand, mutated PHAII genes may exacerbate the inhibition of aldosterone-sensitive renal outer medullary potassium channels (ROMK, a potent potassium secretory channel located in the thick ascending limb of Henle's loop and on the apical membrane of the DCT), worsening the hyperkalemia of GS ( 69 ). NCCT affected by PHAII is the molecular target of thiazide diuretics (six times more sensitive to treatment with thiazides than primary HT individuals), promoting the reversibility of HT and hyperkalemia caused by GS. Angiotensin-converting enzyme inhibitors and angiotensin II receptor blockers are contraindicated in PHA II, as they may worsen hyperkalemia ( 70 ).

## THE BLOOD VESSEL AS A KEY PLAYER ( TABLE 1 ; FIGURE 1 )

### Syndrome of vascular smooth muscle proliferation Hypertension with brachydactyly (Bilginturan syndrome)

Bilginturan syndrome is a rare autosomal dominant disease with high penetrance, characterized by early-onset salt-independent HT, short stature, brachydactyly, and death before 50 years of age, possibly due to stroke ( 71 ). Interestingly, the RAAS and catecholamine secretion are normal. However, vascular or neurovascular abnormalities may suggest that HT can be caused by compression of the ventral-medullary spinal cord, but there is still controversy regarding its pathophysiology. Recent studies have described gain-of-function mutations in the *PDE3A* gene that lead to HT due to increased peripheral vascular resistance ( 72 ). Thus, recognizing this phenotype is essential for diagnosis since the association of short stature, HT in childhood/adolescence, and brachydactyly might suggest Bilginturan syndrome, preventing target organ damage and premature death.

In summary, even though most diseases described above have been considered rare, they may lead to organ damage and increase early mortality risk if not diagnosed and managed correctly. Thus, it is essential to keep in mind and investigate adrenocortical and other monogenic secondary causes of HT, as therapeutic options may be distinct from primary HT. Additionally, knowing the genetic basis of adrenal and other monogenic causes of HT will permit better approaches and promote better patient quality of life.
